# A Remark on the Log-Sobolev Inequality for the Gibbs Measure of the Focusing Schrödinger Equation

**DOI:** 10.1007/s10884-026-10509-y

**Published:** 2026-04-25

**Authors:** Guopeng Li, Jiawei Li, Leonardo Tolomeo

**Affiliations:** 1 School of Mathematics and Statistics, Beijing Institute of Technology, Beijing, 100081 China; 2 School of Mathematics, The University of Edinburgh, and The Maxwell Institute for the Mathematical Sciences, James Clerk Maxwell Building, The King’s Buildings, Edinburgh, EH9 3FD United Kingdom

**Keywords:** Logarithmic Sobolev inequalities, Nonlinear Schrödinger equation, Gibbs measure, 28C20, 35Q55, 60H30

## Abstract

We consider the question of showing a log-Sobolev inequality for the Gibbs measure of the focusing Schrödinger equation built by Lebowitz-Rose-Speer (1988), formally given by

$$\begin{aligned} d\rho \propto \exp \big (\frac{1}{p}\int _{\mathbb T} |u|^p d x - \frac{1}{2}\int _{\mathbb T} |\nabla u|^2 d x - \frac{1}{2}\int _{\mathbb T} |u|^2 d x\big ) 1\!\!1_{\Vert u \Vert _{L^2(\mathbb T)}^2 \le K}dud\overline{u}. \end{aligned}$$

When $2 \le p \le 4$, we show that these measures indeed satisfy a log-Sobolev inequality. When p>4, we establish a lower bound for the Hessian of the effective potential. This implies that the known convexity-based multiscale techniques for the log-Sobolev inequalities cannot be applied to the measure ρ.

## Introduction

In this paper, we consider the focusing Gibbs measures for the nonlinear Schrödinger equations (NLS), initiated in the seminal paper by Lebowitz, Rose, and Speer [26]. A Gibbs measure ρ is a probability measure on functions / distributions whose density can be *formally* given by

1.1 $$\begin{aligned} d\rho = \frac{1}{Z} e^{- H(u)} d u , \end{aligned}$$

where *H*(*u*) is a Hamiltonian functional and *Z* is a normalisation constant, called partition function. In particular, we investigate in the logarithmic Sobolev inequalities (see (1.7)) for the focusing nonlinear Schrödinger equation (NLS) on the circle $\mathbb {T}= \mathbb {R}/ (2\pi \mathbb {Z})$:

1.2 $$\begin{aligned} i u_t + \Delta u + |u|^{p-2} u = 0, \end{aligned}$$

for which the Hamiltonian *H*(*u*) is given by

1.3 $$\begin{aligned} H(u) = \frac{1}{2}\int _\mathbb {T}|\nabla u|^2 d x - \frac{1}{p}\int _\mathbb {T}|u|^p d x. \end{aligned}$$

The study of the equation (1.2) from the viewpoint of the (non-)equilibrium statistical mechanics has received wide attention; see for example [13–18, 20, 25, 26, 34, 35]. See also [9] for a survey on the subject, more from the dynamical point of view.

The main difficulty in constructing the focusing Gibbs measures comes from the high degree p>2 of the negative term in the Hamiltonian (1.3). This makes the problem extremely different from the defocusing case, which would correspond to the well studied $\Phi ^p$ model of quantum field theory. In [26], Lebowitz, Rose, and Speer suggested to consider the Gibbs measure with an extra $L^2$-cutoff

1.4 $$\begin{aligned} \begin{aligned} d\rho&= \frac{1}{Z_K} e^{- H(u) - M(u)} 1\!\!1_{\{M(u) \le K\}} d u \\&= \frac{1}{Z_K} \exp \bigg (\frac{1}{p} \int _\mathbb {T}|u|^p dx\bigg ) 1\!\!1_{\{M(u) \le K\}} d\mu (u), \end{aligned} \end{aligned}$$

where *M*(*u*) denotes the mass functional

1.5 $$\begin{aligned} M(u) = \int _\mathbb {T}|u|^2 dx \end{aligned}$$

and μ is the Gaussian measure given as in (1.6) below. This choice is suitable for the study of the statistical mechanics of NLS, since the mass *M*(*u*) is conserved by the flow of NLS, and represents the “generalised number of particles". In a series of papers [13, 26, 31, 33], the authors showed the following.

### Proposition 1.1

Consider the Gaussian measure

1.6 $$\begin{aligned} d\mu \propto \exp \Big ( - \frac{1}{2} \int _\mathbb {T}|u|^2 dx - \frac{1}{2} \int _\mathbb {T}|\nabla u|^2 dx \Big )dud\overline{u}. \end{aligned}$$

Then the measure ρ in (1.4) is well-defined in the following cases:

1. p<6, K≥0,
2. p=6, and $K \le \Vert Q \Vert _{L^2(\mathbb {R})}^2$, where *Q* is the ground state of the following elliptic equation on $\mathbb {R}$:
  $$\begin{aligned} \Delta Q + Q^5 + 2 Q = 0. \end{aligned}$$

Moreover, we have that in all the cases above, the Radon-Nikodym derivative $f = \frac{d \rho }{d \mu }$ satisfies

1. If p<6, K≥0, $f \in L^q(\mu )$ for every q<∞,
2. If p=6, and $K \le \Vert Q \Vert _{L^2}^2$, then $f \in L^q(\mu )$ for every
  $$\begin{aligned} q \le \Big (\frac{\Vert Q\Vert _{L^2}^2}{K}\Big )^2, \end{aligned}$$
3. If p=6, then for every α<1, $f \in L (\log L)^\alpha$, see [23, Proposition 6].

Here, we focus our attention on the logarithmic Sobolev inequality (LSI) for the Gibbs measures with mass-cutoff. Namely, we ask whether the measure ρ in (1.4) satisfies an inequality of the form

1.7 $$\begin{aligned} \int |F(u)|^2 \log \Big ( \frac{|F(u)|^2}{\int |F(u)|^2 \, d \rho (u)}\Big ) d \rho (u) \;\le \; C \int \Vert \nabla F(u) \Vert _{L^2}^2 \, d \rho (u), \end{aligned}$$

for every suitable smooth functional $F: L^2(\mathbb {T}) \rightarrow \mathbb {R}$ and some constant C>0.

When such an inequality (1.7) holds, we define

1.8 $$\begin{aligned} \textrm{LS}(\rho ) := \inf \Big \{ C>0 \,:\, (1.7) \text { holds for all admissible } F \Big \}, \end{aligned}$$

that is, $\textrm{LS}(\rho )$ is the best (smallest) constant for which (1.7) is valid. If there is no finite constant *C* for which (1.7) holds, we set

1.9 $$\begin{aligned} \textrm{LS}(\rho ) := \infty . \end{aligned}$$

Logarithmic Sobolev inequalities (1.7) are important functional inequalities firstly established by Gross in [21] for Gaussian measures, where its equivalence to hypercontractivity was also shown. LSI is a powerful tool in showing the uniqueness of invariant measure and exponential rate of convergence of the Markov semigroup towards the invariant measure in models from statistical mechanics and quantum field theory, see e.g. [24, 37, 38]. See [2, 22] for comprehensive reviews.

In [1], Bakry and Émery provide a nice sufficient condition for LSI under log-concave measures. For a Gibbs measure ρ as in (1.1), Bakry-Émery criterion (see Proposition 2.1) implies LSI holds when the Hamiltonian *H* is strictly convex. For applications of Bakry-Émery criterion, see [19, 22, 27].

In the context of Gibbs measures for NLS, [10] was, to the best of our knowledge, the first work attempting to address a logarithmic Sobolev inequality for the focusing NLS with $2 \le p \le 4$ via the Bakry-Émery criterion. We however point out that in [10] the authors seem to overlook the complications deriving from the presence of the cutoff function. More specifically, the authors first prove that an appropriate potential is convex on the ball, and then refer to the result in [1] to conclude the log-Sobolev inequality. However, the result in [1] does not apply to this very general setting. Indeed, if instead the cutoff function in (1.4) is put on a non-convex subset of the ball, then the log-Sobolev inequality (1.7) might fail (counterexamples can be constructed easily when the set is disconnected and *F* is constant on the connected components), so a certain level of care around the cutoff is necessary.

In our first main result we solve these issues, and show the following.

### Theorem 1.2

Let $2 \le p \le 4$ and K>0, and let ρ be the focusing Gibbs measure with $L^2$-cutoff at level *K* given in (1.4), associated with the nonlinear Schrödinger equation (1.2) on $\mathbb {T}$. Then ρ satisfies a logarithmic Sobolev inequality of the form (1.7). In particular,

$$ \textrm{LS}(\rho ) < \infty , $$

in the sense of definition (1.8).

### Remark 1.3

By [22, Theorem 4.9], and Lemma 3.1, the log-Sobolev inequality proven in Theorem 1.2 implies uniqueness of the invariant measure and spectral gap for an appropriately defined Markov process. This process can be characterised as the limit as $R\rightarrow \infty$ of the Markov process obtained by solving the SPDE

$$\begin{aligned} \partial _t u + (1 -\Delta ) u = |u|^{p-2}u -16 R (\Vert u\Vert _{L^2}^2 - K)_+^7 u + 2\xi , \end{aligned}$$

where ξ is a complex-valued space-time white noise.

In analogy to the finite dimensional setting, we expect that this Markov process can also be given by the solution to the reflected SPDE

$$\begin{aligned} \partial _t u + (1 -\Delta ) u = |u|^{p-2}u - K^{-\frac{1}{2}} u \dot{L}_t + 2\xi , \end{aligned}$$

where $L_t$ denotes the local time of *u* at the boundary $\{ \Vert u \Vert _{L^2}^2 = K \}$. However, showing the equivalence of the two Markov processes is outside the scope of this paper.

When p>4, the situation is more complex. In general, establishing LSI beyond the strict convexity assumption on *H* is challenging, especially on infinite-dimensional spaces. In recent years, there have been several breakthroughs by Bauerschmidt, Bodineau, and collaborators, see [4–7], that culminated with the proof of uniqueness for the $\varphi ^4_2$ measure in high temperature in [8]. These results have been obtained via the use of a “multiscale Bakry-Émery formula", see [5, Theorem 2.5], that we now describe in the particular case of the measure ρ. Define the family of Gaussian measures $\bar{\mu }_t$ *formally* given by

$$\begin{aligned} d\bar{\mu }_t (u) \propto \exp \Big (-\frac{1}{2} \langle u, \frac{\langle \nabla \rangle ^2}{1-e^{-t \langle \nabla \rangle ^2}}u \rangle \Big ) dud\overline{u}. \end{aligned}$$

Note that if *u* is a random variable with ${{\,\textrm{Law}\,}}(u) = \mu$ in (1.6), then

1.10 $$\begin{aligned} {{\,\textrm{Law}\,}}( \sqrt{1 - e^{-t\langle \nabla \rangle ^2}} u) = \bar{\mu }_t. \end{aligned}$$

We then set

$$\begin{aligned} V_t(\varphi ) = \log \Big (\mathbb {E}_{\bar{\mu }_t} \Big [ \exp \Big (\frac{1}{p} \int _\mathbb {T}|u+ \varphi |^p dx\Big )1\!\!1_{ \Vert u + \varphi \Vert _{L^2}^2 \le K} \Big ]\Big ) \end{aligned}$$

for every $\varphi \in H^1$ (note that our $V_t$ is the negative of the renormalized potential defined in [5]). Theorem 2.5 in [5] states that under some suitable ergodicity assumption, if for every t≥0 there exists a differentiable map $t \mapsto \lambda _t \in \mathbb {R}$ such that

$$\begin{aligned} \int _0^\infty e^{-2 \lambda _t} dt < \infty \end{aligned}$$

and the Hessian of $V_t(\varphi )$ denoted by $\mathcal {H}(V_t)(\varphi )$ satisfies

1.11 $$\begin{aligned} \mathcal {H}(V_t)(\varphi )[e^{-\frac{t\langle \nabla \rangle ^2}{2}}w, e^{-\frac{t\langle \nabla \rangle ^2}{2}}w] \le \frac{1}{2} \Vert w \Vert _{H^1}^2 - \dot{\lambda }_t \Vert w \Vert _{L^2}^2 \end{aligned}$$

for all $w\in H^1$, then LSI holds.

Instead of working with (1.11), we consider the functional

1.12 $$\begin{aligned} V(\varphi ) = \log \Big (\mathbb {E}_\mu \Big [ \exp \Big (\frac{1}{p} \int _\mathbb {T}|u+ \varphi |^p dx\Big )1\!\!1_{ \Vert u + \varphi \Vert _{L^2}^2 \le K} \Big ]\Big ), \end{aligned}$$

where μ is as in (1.6). Note that, abusing of notation, we have $V = V_\infty$.

By taking *t* large in (1.11), by boundedness of the operator $\sqrt{1-e^{-t\langle \nabla \rangle ^2}}$ and its inverse, due to the integrability condition for $\lambda _t$, in order to apply this criterion we essentially require that, for $w \in H^1$,

1.13 $$\begin{aligned} \sup _{\varphi \in H^1} \mathcal {H}(V)(\varphi )[w,w] < \infty . \end{aligned}$$

We define the probability measure

1.14 $$\begin{aligned} d\rho _\varphi (u) = \frac{1}{Z_\varphi } \exp \Big (\frac{1}{p} \int _\mathbb {T}|u+ \varphi |^p dx\Big )1\!\!1_{ \Vert u + \varphi \Vert _{L^2}^2 \le K} d\mu (u), \end{aligned}$$

where $Z_\varphi$ is an appropriate normalisation constant.

The second main result of this paper is the following.

### Theorem 1.4

Let 4<p<6 and let *V* be defined by (1.12), with $\rho _\varphi$ the probability measures given in (1.14). Then the following hold:

(i) For every $\varphi \in H^1(\mathbb {T})$ and every $w \in H^1(\mathbb {T})$,

1.15 $$\begin{aligned} \mathcal {H}V(\varphi )[w,w] \;\ge \; (p-1)\int _{\mathbb {T}} \mathbb {E}_{\rho _\varphi }\big [\,|u(x)|^{p-2}\,\big ]\, |w(x)|^2\,dx. \end{aligned}$$

(ii) We have

1.16 $$\begin{aligned} \sup _{\varphi \in H^1(\mathbb {T})} \mathbb {E}_{\rho _\varphi }\Big [\int _{\mathbb {T}} |u(x)|^{p-2}\,dx\Big ] \;=\; \infty . \end{aligned}$$

In particular, for every $w \in H^1(\mathbb {T})\setminus \{0\}$,

1.17 $$\begin{aligned} \sup _{\varphi \in H^1(\mathbb {T})} \mathcal {H}V(\varphi )[w,w] \;=\; \infty . \end{aligned}$$

Theorem 1.4 tells us that when 4<p<6, the Hessian $\mathcal {H}V(\varphi )$ cannot be uniformly bounded from above on $H^1(\mathbb {T})$ in any fixed direction w≠0. Consequently, the multiscale Bakry-Émery criterion from [5] cannot be applied to establish an LSI for the associated Gibbs measure. We emphasize that this result demonstrates an inherent structural obstruction to the current multiscale convexity-based methods, indicating the failure of this specific approach. It does *not* imply the failure of the logarithmic Sobolev inequality itself, which remains an open question in the super-quartic regime.

## Preliminaries

Throughout the paper we work with complex-valued functions $u:\mathbb {T}\rightarrow \mathbb {C}$ and view $L^2(\mathbb {T};\mathbb {C})=L^2(\mathbb {T})$ as a real Hilbert space with scalar product

$$ \langle f,g\rangle := \mathop {\textrm{Re}}\limits \int _{\mathbb {T}} f(x)\,\overline{g(x)}\,dx. $$

In particular, $\Vert u\Vert _{L^2}^2 = \langle u,u\rangle$ and all gradients and Hessians are taken with respect to this real structure.

We use C>0 to denote various constants, which may vary line by line. We also use A≲B to denote an estimate of the form A≤CB for some constant C>0. We denote by *dx* the Lebesgue measure on the torus, normalised so that $\int _\mathbb {T}dx = 1$.

Given $N \in \mathbb {N}$, we denote by $P_N$ the projector onto the frequencies $\{|n| \le N\}$ defined by

$$\begin{aligned} \widehat{P_N f}(n)= 1\!\!1_{\{|n|\le N\}} \widehat{f}(n). \end{aligned}$$

We remark that due to the boundedness of the Hilbert transform on the torus, we have that

2.1 $$\begin{aligned} \Vert P_N f - f \Vert _{L^p} \rightarrow 0 \end{aligned}$$

as $N\rightarrow \infty$, for every $f \in L^p$, 1<p<∞.

Finally, we set $x_+ = \max \{x,0\}$.

To show Theorem 1.2, we will use the following celebrated criterion.

### Proposition 2.1

(Bakry-Émery [1]) Consider a probability measure on $\mathbb {R}^n$ (or a linear sub-space) of the form

$$\begin{aligned} d\nu (x) \propto e^{-H(x)}dx, \end{aligned}$$

and assume that there is λ>0 such that as quadratic forms:

2.2 $$\begin{aligned} \mathcal {H}(H(x)) \ge \lambda \cdot \textrm{id}. \end{aligned}$$

Then, we have

$$\begin{aligned} \textrm{LS}(\nu ) \le \frac{2}{\lambda } < \infty . \end{aligned}$$

### Gaussian Measure and Gibbs Measure

The Gaussian measure μ we introduced formally in (1.6) can be defined rigorously as the law of

2.3 $$\begin{aligned} u(x) = \sum _{n\in \mathbb {Z}} \frac{g_n}{\langle n \rangle }e^{inx}, \end{aligned}$$

where $\{g_n\}_{n\in \mathbb {Z}}$ is a family of mutually independent complex-valued centred Gaussian random variables. By standard bounds on Gaussian random variables, we have the following.

#### Lemma 2.2

Let *u* be a function-valued random variable with ${{\,\textrm{Law}\,}}(u) = \mu$ in (1.6). Then for every q<∞, and every $x \in \mathbb {T}$, we have that

2.4 $$\begin{aligned} \mathbb {E}|u(x)|^q = \mathbb {E}\Vert u \Vert _{L^q(\mathbb {T})}^q < \infty . \end{aligned}$$

Moreover, using (2.3), we can deduce the following fact.

#### Lemma 2.3

Let K>0, and consider the set

$$\begin{aligned} S_K = \{ u \in L^2(\mathbb {T}): \Vert u \Vert _{L^2}^2 = K\}. \end{aligned}$$

Then

$$\begin{aligned} \mu (S_K) = 0. \end{aligned}$$

#### Proof

The proof is completely analogous to [32, Lemma 2.7]. □

### Boué-Dupuis Variational Formula

In order to state the Boué-Dupuis variational formula, as in [3, 28–30, 33], we first need to introduce some notations. Let $W_t$ be a cylindrical Brownian motion in $L^2(\mathbb {T})$ given by

$$\begin{aligned} W_t = \sum _{n \in \mathbb {Z}} B_n(t) e^{inx}, \end{aligned}$$

where $\{B_n\}_{n \in \mathbb {Z}}$ is a family of mutually independent complex-valued^1^ Brownian motions on a probability space $(\Omega , \mathcal {F}, \mathbb {P})$. Let $(\mathcal {F}_t)_{t\in [0, 1]}$ be the $\mathbb {P}$-completion of the filtration generated by $(W_t)_{t\in [0,1]}$. We then define a centered Gaussian process $Y_t$ by

2.5 $$\begin{aligned} Y_t = \langle \nabla \rangle ^{-1}W_t. \end{aligned}$$

Note that we have $${\mathbb {P}}\circ (Y_1)^{-1} = \mu $$. By setting $$Y^N = P_N Y $$, we have $$\mathbb {P}\circ (Y^N_1)^{-1} = (P_N)_*\mu $$, i.e. the push-forward of μ under $$P_N$$.

Next, let $\mathbb {H}_a$ denote the space of drifts, containing processes that are progressively measurable with respect to $(\mathcal {F}_t)_{t\in [0,1]}$, belonging to $\dot{H}^1([0,1]; H^1(\mathbb {T}))$, and which are 0 in 0. We now state the variational formula [12, 36]; in particular, see Theorem 7 in [36].

#### Lemma 2.4

(Boué-Dupuis variational formula) Let *Y* be as in (2.5) and fix $N \in \mathbb {N}$. Suppose that $V:C^\infty (\mathbb {T}) \rightarrow \mathbb {R}$ is measurable, and satisfies $\mathbb {E}\big [|V(P_NY_1)|^p\big ] < \infty$ and $\mathbb {E}\big [e^{p'V(P_NY_1)} \big ] < \infty$, for some 1<p<∞, with

$$\begin{aligned} \frac{1}{p} + \frac{1}{p'} = 1. \end{aligned}$$

Then, we have

2.6 $$\begin{aligned} \log \mathbb {E}\Big [e^{V(P_N Y_1)}\Big ] = \sup _{\Theta \in \mathbb {H}_a} \mathbb {E}\bigg [ V(P_N Y_1 + P_N \Theta (1)) - \frac{1}{2} \int _0^1 \Vert \dot{\Theta }(t) \Vert _{H^1_x}^2 dt \bigg ], \end{aligned}$$

where the expectation $\mathbb {E}= \mathbb {E}_\mathbb {P}$ is with respect to the underlying probability measure $\mathbb {P}$.

The following consequence of this formula will play a fundamental role.

#### Lemma 2.5

Let *Y*, *N*, *V* be as in Lemma 2.4. Let 0<ε<1, and suppose that $\Theta _\varepsilon \in \mathbb {H}_a$ is an $\varepsilon ^2$-optimiser for the expression in (2.6), in the sense that

$$\begin{aligned} \mathbb {E}\bigg [&V(P_N Y_1 + P_N \Theta _\varepsilon (1)) - \frac{1}{2} \int _0^1 \Vert \dot{\Theta }_\varepsilon (t) \Vert _{H^1_x}^2 dt \bigg ] \\&\ge -\varepsilon ^2 + \sup _{\Theta \in \mathbb {H}_a} \mathbb {E}\bigg [ V(P_N Y_1 + P_N \Theta (1)) - \frac{1}{2} \int _0^1 \Vert \dot{\Theta }(t) \Vert _{H^1_x}^2 dt \bigg ]. \end{aligned}$$

Let $F: C^\infty (\mathbb {T}) \rightarrow \mathbb {R}$ be measurable and bounded. Then, we have that

2.7 $$\begin{aligned} \Big |\frac{\mathbb {E}[F(P_N Y_1)\exp (V(P_N Y_1))]}{\mathbb {E}[\exp (V(P_NY_1))]} - \mathbb {E}[F(P_N (Y_1 + \Theta _\varepsilon (1)))] \Big | \le (1 + \frac{e}{2})\varepsilon \Vert F \Vert _{L^\infty } \end{aligned}$$

#### Proof

Since the map

$$ F \mapsto \frac{\mathbb {E}[F(P_N Y_1)\exp (V(P_N Y_1))]}{\mathbb {E}[\exp (V(P_NY_1))]} $$

defines a probability measure, it is enough to show (2.7) when $F = 1\!\!1_E$ is an indicator function. Moreover, by swapping *E* with $E^c$, it is enough to show that

2.8 $$\begin{aligned} \frac{\mathbb {E}[1\!\!1_E(P_N Y_1)\exp (V(P_N Y_1))]}{\mathbb {E}[\exp (V(P_NY_1))]} - \mathbb {E}[1\!\!1_E(P_N (Y_1 + \Theta _\varepsilon (1)))]\ge -(1+\frac{e}{2})\varepsilon . \end{aligned}$$

For this, we apply (2.6) with *V* replaced by $\varepsilon 1\!\!1_E + V$, for $\varepsilon \ge 0$. By the elementary inequalities

$$ e^{\varepsilon } \le 1 + \varepsilon + \frac{e}{2} \varepsilon ^2 \quad \quad \text {and} \quad \quad \log (1 + x) \le x $$

for $0\le \varepsilon \le 1$, x≥0, we have that

$$\begin{aligned} \big (\varepsilon + \tfrac{e}{2} \varepsilon ^2\big )&\frac{\mathbb {E}\big [1\!\!1_E(P_N Y_1)\,e^{V(P_N Y_1)}\big ]}{\mathbb {E}\big [e^{V(P_NY_1)}\big ]} \\&\ge \log \frac{\mathbb {E}\big [e^{(\varepsilon 1\!\!1_E + V)(P_N Y_1)}\big ]}{\mathbb {E}\big [e^{V(P_NY_1)}\big ]} \\&= \sup _{\Theta \in \mathbb {H}_a} \mathbb {E}\bigg [ (\varepsilon 1\!\!1_E + V)\big (P_N Y_1 + P_N \Theta (1)\big ) - \frac{1}{2} \int _0^1 \Vert \dot{\Theta }(t)\Vert _{H^1_x}^2 \, dt \bigg ] \\&\phantom {=} \;-\; \sup _{\Theta \in \mathbb {H}_a} \mathbb {E}\bigg [ V\big (P_N Y_1 + P_N \Theta (1)\big ) - \frac{1}{2} \int _0^1 \Vert \dot{\Theta }(t)\Vert _{H^1_x}^2 \, dt \bigg ] \\&\ge \mathbb {E}\bigg [ (\varepsilon 1\!\!1_E + V)\big (P_N Y_1 + P_N \Theta _\varepsilon (1)\big ) - \frac{1}{2} \int _0^1 \Vert \dot{\Theta }_\varepsilon (t)\Vert _{H^1_x}^2 \, dt \bigg ] \\&\phantom {\ge }\; -\,\varepsilon ^2 \;-\; \mathbb {E}\bigg [ V\big (P_N Y_1 + P_N \Theta _\varepsilon (1)\big ) - \frac{1}{2} \int _0^1 \Vert \dot{\Theta }_\varepsilon (t)\Vert _{H^1_x}^2 \, dt \bigg ] \\&= -\,\varepsilon ^2 + \varepsilon \,\mathbb {E}\Big [1\!\!1_E\big (P_N (Y_1 + \Theta _\varepsilon (1))\big )\Big ]. \end{aligned}$$

from which we deduce

$$ \frac{\mathbb {E}[1\!\!1_E(P_N Y_1)\exp (V(P_N Y_1))]}{\mathbb {E}[\exp (V(P_NY_1))]} - \mathbb {E}[1\!\!1_E(P_N (Y_1 + \Theta _\varepsilon (1)))] \;\ge \; -\Big (1 + \frac{e}{2}\Big ) \varepsilon , $$

and hence (2.7).

□

## Proof of Theorem 1.2

Throughout this section, we consider the measure

3.1 $$\begin{aligned} d\rho _{\Lambda }(u) = \frac{1}{Z_\Lambda } \exp \Big (- \Lambda \int _\mathbb {T}|u|^2 dx\Big ) d \rho (u), \end{aligned}$$

where $Z_\Lambda$ is an appropriate normalisation constant for a given parameter Λ>0. Since ρ is supported on the set $\{ \Vert u \Vert _{L^2}^2 \le K \}$, we have that

3.2 $$\begin{aligned} \exp (-\Lambda K) \textrm{LS}(\rho ) \le \textrm{LS}(\rho _\Lambda ) \le \exp ( \Lambda K) \textrm{LS}(\rho ). \end{aligned}$$

We have the following lemma.

### Lemma 3.1

Let p<6, and let $N \in \mathbb {N}$, R>0 and Λ>0. Consider the measures

$$\begin{aligned} d&\rho _{\Lambda ,N,R} \\&= \frac{1}{Z_{\Lambda ,N,R}} \exp \Big ( - \Lambda \int _\mathbb {T}|P_Nu|^2 dx + \frac{1}{p} \int _\mathbb {T}|P_Nu|^p dx- R(\Vert P_Nu\Vert _{L^2}^2 - K)_+^8 \Big ) d \mu . \end{aligned}$$

Then we have that

$$\begin{aligned} \lim _{N \rightarrow \infty } \lim _{R \rightarrow \infty } \rho _{\Lambda , N, R} = \rho _\Lambda , \end{aligned}$$

where $\rho _\Lambda$ is given as in (3.1) and the limits are taken in total variation.

### Proof

It is enough to show that

$$\begin{aligned} \lim _{N \rightarrow \infty }&\lim _{R \rightarrow \infty } \exp \Big ( - \Lambda \int _\mathbb {T}|P_Nu|^2 dx + \frac{1}{p} \int _\mathbb {T}|P_Nu|^p dx - R(\Vert P_Nu\Vert _{L^2}^2 - K)_+^8 \Big ) \\&\quad \,= \exp \Big ( -\Lambda \int _\mathbb {T}|u|^2 dx + \frac{1}{p} \int _\mathbb {T}|u|^p dx\Big ) 1\!\!1_{\Vert u\Vert _{L^2}^2 \le K} \end{aligned}$$

in $L^1(\mu )$. We first check pointwise convergence and then verify a uniform integrability bound.

We have that

$$\begin{aligned} \lim _{N \rightarrow \infty }&\lim _{R \rightarrow \infty } \exp \Big ( - \Lambda \int _\mathbb {T}|P_Nu|^2 dx + \frac{1}{p} \int _\mathbb {T}|P_Nu|^p dx - R(\Vert P_Nu\Vert _{L^2}^2 - K)_+^8 \Big ) \\&\quad \,= \exp \Big ( -\Lambda \int _\mathbb {T}|u|^2 dx + \frac{1}{p} \int _\mathbb {T}|u|^p dx\Big ) 1\!\!1_{\Vert u\Vert _{L^2}^2 \le K} \end{aligned}$$

pointwise, so it is enough to show that for any q>1,

3.3 $$\begin{aligned} \sup _{N \in \mathbb {N}} \limsup _{R \rightarrow \infty } \int \exp \Big ( \frac{q}{p} \int _\mathbb {T}|P_Nu|^p - qR(\Vert P_Nu\Vert _{L^2}^2 - K)_+^8 \Big ) d\mu (u) < \infty . \end{aligned}$$

By dominated convergence theorem, we have that

$$\begin{aligned} \sup _{N \in \mathbb {N}}&\limsup _{R \rightarrow \infty } \mathbb {E}_\mu \bigg [ \exp \Big ( \frac{q}{p} \int _\mathbb {T}|P_Nu|^p dx - qR(\Vert P_Nu\Vert _{L^2}^2 - K)_+^8 \Big ) \bigg ] \\&\,\,= \sup _{N \in \mathbb {N}} \mathbb {E}_\mu \bigg [ \exp \Big ( \frac{q}{p} \int _\mathbb {T}|P_Nu|^p dx \Big )1\!\!1_{\Vert P_Nu \Vert _{L^2}^2 \le K} \bigg ], \end{aligned}$$

which is finite by [33, Proposition 3.1]. □

### Proof of Theorem 1.2

By (3.2), it suffices to show that for Λ big enough, $\textrm{LS}(\rho _\Lambda ) <\infty$. Moreover, it is enough to show the inequality (1.7) for bounded cylindrical functions *F*, in the sense that there exists $N_0 \in \mathbb {N}$ such that $F(u) = F(P_{N_0} (u))$. Finally, in view of Lemma 3.1, the result follows once we show that

$$\begin{aligned} \textrm{LS}((P_N)_* \rho _{\Lambda ,N,R}) \lesssim 1 \end{aligned}$$

uniformly in *N* and *R*. We have that, on the image of $P_N$ (which is finite dimensional, and can be identified with $\mathbb {C}^{2N+1}$), the measure $(P_N)_* \rho _{\Lambda ,N,R}$ is of the form

$$\begin{aligned}&d(P_N)_* \rho _{\Lambda ,N,R}(u) \\&\qquad \propto \exp \Big (- \Lambda \int _\mathbb {T}|u|^2 dx + \frac{1}{p} \int _\mathbb {T}|u|^p dx - R(\Vert u\Vert _{L^2}^2 - K)_+^8 - \frac{1}{2} \Vert u \Vert _{H^1}^2 \Big ) du d\overline{u}. \end{aligned}$$

Therefore, we can apply Proposition 2.1 to

$$\begin{aligned} H(u) = \Lambda \int _\mathbb {T}|u|^2 dx - \frac{1}{p} \int _\mathbb {T}|u|^p dx + R(\Vert u\Vert _{L^2}^2 - K)_+^8 + \frac{1}{2} \Vert u \Vert _{H^1}^2. \end{aligned}$$

We have that

3.4 $$\begin{aligned} \begin{aligned} \mathcal {H}H(u)&= 2 \Lambda \cdot \textrm{id}- (p-1) |u|^{p-2} + 16R (\Vert u\Vert _{L^2}^2 - K)_+^7 \cdot \textrm{id}\\&\phantom {=\ }+ 224R (\Vert u\Vert _{L^2}^2 - K)_+^6 u \otimes u + (1-\Delta ), \end{aligned} \end{aligned}$$

which implies that

$$\begin{aligned} \begin{aligned} \mathcal {H} H(u)&\ge 2\Lambda \cdot \textrm{id} - (p-1)|u|^{p-2} + 16R(\Vert u\Vert _{L^2}^2-K)_+^7 \cdot \textrm{id} + (1-\Delta ) \end{aligned} \end{aligned}$$

as operators.

For $w \in H^1$, since p-2≤2, by Sobolev embeddings and Young’s inequality, we have that (for some constant C>0 that can change from line to line)

$$\begin{aligned} (p-1) \int _\mathbb {T}|u|^{p-2}|w|^2 dx&\le \int _\mathbb {T}C(1 + |u|^2)|w|^2 dx\\&\le C(1 + \Vert u\Vert _{L^2}^2) \Vert w \Vert _{L^\infty }^2 \\&\le C(1 + \Vert u\Vert _{L^2}^2) \Vert w \Vert _{H^\frac{3}{4}}^ 2 \\&\le C(1 + \Vert u\Vert _{L^2}^2) \Vert w \Vert _{L^2}^{\frac{1}{2}} \Vert w \Vert _{H^1}^{\frac{3}{2}} \\&\le C(1 + \Vert u\Vert _{L^2}^2)^4 \Vert w \Vert _{L^2}^2 + \frac{1}{2} \Vert w \Vert _{H^1}^2. \end{aligned}$$

This shows that

$$\begin{aligned} (p-1) |u|^{p-2}\le C(1 + \Vert u\Vert _{L^2}^2)^4 \cdot \textrm{id}+ (1-\Delta ) \end{aligned}$$

as operators. Moreover, there exists a constant $\Lambda _* = \Lambda _*(K)$ such that

$$\begin{aligned} (\Vert u\Vert _{L^2}^2 - K)_+^7 - C(1 + \Vert u\Vert _{L^2}^2)^4 \ge - \Lambda _*. \end{aligned}$$

Therefore, for $R > \frac{1}{16}$, we get that as operators

$$\begin{aligned} \mathcal {H}H(u)&\ge (2 \Lambda - \Lambda _*)\cdot \textrm{id}\ge \textrm{id}\end{aligned}$$

as long as $\Lambda \ge \frac{\Lambda _* + 1}{2}$, which shows that

$$\begin{aligned} \textrm{LS}((P_N)_* \rho _{\Lambda ,N,R}) \le 2. \end{aligned}$$

□

## Proof of Theorem 1.4

The proof of Theorem 1.4 is heavily reliant on the description of the measure $\rho _\varphi$ provided by Lemma 2.5. To see this, we first show the following lower bound on the quantity $\mathcal {H}V(\varphi )$, which is (1.15) in Theorem 1.4.

### Proposition 4.1

Let V(φ) be given as in (1.12). Then for all $\varphi \in H^1(\mathbb {T})$, we have that

4.1 $$\begin{aligned} \mathcal {H}(V)(\varphi )[w,w] \ge (p-1)\int _\mathbb {T}\mathbb {E}_{\rho _\varphi }[|u|^{p-2}] |w|^2 dx. \end{aligned}$$

### Proof

By Cameron-Martin’s theorem (see e.g. [11, Corollary 2.4.3]), we may write that

$$\begin{aligned} V(\varphi ) = \log \bigg (\mathbb {E}_\mu \bigg [ \exp \Big ( \frac{1}{p} \int _\mathbb {T}|u|^ p dx + \langle u, (1-\Delta ) \varphi \rangle -\frac{1}{2} \Vert \varphi \Vert _{H^1}^2 \Big ) 1\!\!1_{\Vert u\Vert _{L^2}^2 \le K}\bigg ]\bigg ). \end{aligned}$$

In particular, we have that *V* is smooth as a function of φ, and by computation similar to (3.4) and the argument in the proof of Lemma 3.1, we have

$$\begin{aligned} \mathcal {H}V(\varphi ) = \lim _{R \rightarrow \infty } \mathcal {H}V_{R} (\varphi ), \end{aligned}$$

where

$$ V_{R}(\varphi ) = \log \bigg (\mathbb {E}_\mu \bigg [ \exp \Big (\frac{1}{p} \int _\mathbb {T}|u+ \varphi |^p dx -R \big (\Vert u + \varphi \Vert _{L^2}^2 - K\big )_+^{\sigma }\Big )\bigg ]\bigg ),$$

and $\sigma > \frac{p}{2} + 1$. For an appropriately integrable functional F(φ), by the chain rule we have that

$$\begin{aligned} \nabla \log \big (\mathbb {E}_\mu [\exp (F(\varphi ))]\big ) = \frac{\mathbb {E}_\mu [\nabla F \exp (F(\varphi ))]}{\mathbb {E}_\mu [\exp (F(\varphi ))]} \end{aligned}$$

and so

$$\begin{aligned}&\mathcal {H}\log \big (\mathbb {E}_\mu [\exp (F(\varphi ))]\big )[w,w] \\&= \frac{\mathbb {E}_\mu [\mathcal {H}F[w,w] \exp (F(\varphi ))]}{\mathbb {E}_\mu [\exp (F(\varphi ))]} + \frac{\mathbb {E}_\mu [|\nabla F \cdot w|^2 \exp (F(\varphi ))]}{\mathbb {E}_\mu [\exp (F(\varphi ))]} \\&\quad - \Big | \frac{\mathbb {E}_\mu [\nabla F \cdot w \exp (F(\varphi ))]}{\mathbb {E}_\mu [\exp (F(\varphi ))]} \Big |^2 \\&\ge \frac{\mathbb {E}_\mu [\mathcal {H}F[w,w] \exp (F(\varphi ))]}{\mathbb {E}_\mu [\exp (F(\varphi ))]}, \end{aligned}$$

where we used Jensen’s inequality for probability measure

$$\begin{aligned} \frac{d\nu }{d\mu } = \frac{\exp (F(\varphi ))}{\mathbb {E}_\mu [\exp (F(\varphi ))]} \end{aligned}$$

in the last step. Applying to $V_R$, we get

$$\begin{aligned}&\mathcal {H}(V_R)(\varphi )[w,w] \\&\ge (p-1) \frac{\mathbb {E}_\mu \Big [ \big ( \int _\mathbb {T}|u+\varphi |^{p-2} |w|^2 \big ) \exp \Big (\frac{1}{p} \int _\mathbb {T}|u+ \varphi |^p-R \big (\Vert u + \varphi \Vert _{L^2}^2 - K\big )_+^{\sigma }\Big ) \Big ]}{\mathbb {E}_\mu \Big [ \exp \Big (\frac{1}{p} \int _\mathbb {T}|u+ \varphi |^p-R \big (\Vert u + \varphi \Vert _{L^2}^2 - K\big )_+^{\sigma }\Big ) \Big ]}\\&\phantom {\ge \ }- \frac{\mathbb {E}_\mu \Big [ \big ( R \mathcal {H}\big (\Vert u + \varphi \Vert _{L^2}^2 - K\big )_+^{\sigma } [w,w] \big ) \exp \Big (\frac{1}{p} \int _\mathbb {T}|u+ \varphi |^p-R \big (\Vert u + \varphi \Vert _{L^2}^2 - K\big )_+^{\sigma }\Big ) \Big ]}{\mathbb {E}_\mu \Big [ \exp \Big (\frac{1}{p} \int _\mathbb {T}|u+ \varphi |^p-R \big (\Vert u + \varphi \Vert _{L^2}^2 - K\big )_+^{\sigma }\Big ) \Big ]}. \end{aligned}$$

By taking limits as $R \rightarrow \infty ,$ we deduce

$$\begin{aligned}&\mathcal {H}(V)(\varphi )[w,w] \\&\ge (p-1) \frac{\mathbb {E}\Big [ \big ( \int _\mathbb {T}|u+\varphi |^{p-2} |w|^2 \big ) \exp \Big (\frac{1}{p} \int _\mathbb {T}|u+ \varphi |^p\Big )1\!\!1_{ \Vert u + \varphi \Vert _{L^2}^2 \le K} \Big ]}{\mathbb {E}\Big [ \exp \Big (\frac{1}{p} \int _\mathbb {T}|u+ \varphi |^p\Big )1\!\!1_{ \Vert u + \varphi \Vert _{L^2}^2 \le K} \Big ]}, \end{aligned}$$

which is (4.1).

□

### Lemma 4.2

Let 2≤p<6. For every $\varphi \in H^1(\mathbb {T})$, we have that $V(\varphi ) < \infty .$ Moreover, for $N \in \mathbb {N}\cup \{ \infty \}$ dyadic, L>0, define $\rho _{N,L,\varphi }$ to be the probability measure

4.2 $$\begin{aligned} \begin{aligned} d \rho _{N,L,\varphi } = \frac{1}{Z_{{N,L,\varphi }}} \exp \Big ( \frac{1}{p} \int _\mathbb {T}|P_N(u+\varphi )|^p dx 1\!\!1_{\Vert P_N(u+\varphi )\Vert _{L^2}^2 \le K} \\ \hspace{100pt}- L 1\!\!1_{\Vert P_N(u+\varphi )\Vert _{L^2}^2 > K}\Big ) d \mu , \end{aligned} \end{aligned}$$

where $Z_{{N,L,\varphi }}$ is an appropriate normalisation constant. Then for every q<∞, we have that

4.3 $$\begin{aligned} \lim _{N \rightarrow \infty } \mathbb {E}_{\rho _{N,L,\varphi }}\Big [ 1\!\!1_{\Vert u\Vert _{L^2}^2 \le K} \int _\mathbb {T}|u|^q dx \Big ] = \mathbb {E}_{\rho _{\infty ,L,\varphi }}\Big [ 1\!\!1_{\Vert u\Vert _{L^2}^2 \le K} \int _\mathbb {T}|u|^q dx \Big ], \end{aligned}$$

and

4.4 $$\begin{aligned} V(\varphi ) = \lim _{N \rightarrow \infty , L \rightarrow \infty } \log Z_{{N,L,\varphi }}. \end{aligned}$$

### Proof

By (2.1), we have that for every 1<p<∞,

$$\begin{aligned} P_N(u + \varphi ) \rightarrow u + \varphi \end{aligned}$$

in $L^p(\mathbb {T})$. Therefore, from Lemma 2.3, we have that

$$\begin{aligned} \begin{aligned} \exp \Big (\frac{1}{p} \int _\mathbb {T}|P_N(u+ \varphi )|^p dx 1\!\!1_{ \Vert P_N(u+ \varphi ) \Vert _{L^2}^2 \le K} - L 1\!\!1_{ \Vert P_N(u+ \varphi ) \Vert _{L^2}^2> K}\Big ) \\ \rightarrow \exp \Big (\frac{1}{p} \int _\mathbb {T}|u+ \varphi |^p dx 1\!\!1_{ \Vert u+ \varphi \Vert _{L^2}^2 \le K}- L 1\!\!1_{ \Vert u+ \varphi \Vert _{L^2}^2 > K}\Big ) \end{aligned} \end{aligned}$$

μ-a.s. as $N \rightarrow \infty$, and also from Lemma 2.3, we have

$$\begin{aligned} \begin{aligned} \exp \Big (\frac{1}{p} \int _\mathbb {T}|P_N(u+ \varphi )|^p dx 1\!\!1_{ \Vert P_N(u+ \varphi ) \Vert _{L^2}^2 \le K} - L 1\!\!1_{ \Vert P_N(u+ \varphi ) \Vert _{L^2}^2 > K}\Big ) \\ \rightarrow \exp \Big (\frac{1}{p} \int _\mathbb {T}|u+ \varphi |^p dx \Big ) 1\!\!1_{ \Vert u+ \varphi \Vert _{L^2}^2 \le K} \end{aligned} \end{aligned}$$

μ-a.s. as $N,L \rightarrow \infty$. Recalling that by Lemma 2.2, we have that

$$\begin{aligned} \mathbb {E}_{\mu } [|u|^q] \lesssim _q 1 \end{aligned}$$

for every q<∞, for both (4.3) and (4.4), it is enough to show that for any q>1,

$$\begin{aligned} \sup _{N,L \in \mathbb {N}}&\mathbb {E}_{\mu }\Big [ \exp \Big (\frac{q}{p} \int _\mathbb {T}|P_N(u+ \varphi )|^p dx 1\!\!1_{ \Vert P_N(u+ \varphi ) \Vert _{L^2}^2 \le K}- qL 1\!\!1_{ \Vert P_N(u+ \varphi ) \Vert _{L^2}^2 > K}\Big )\Big ) \Big ] \\&\le \sup _{N \in \mathbb {N}} \mathbb {E}_{\mu }\Big [ \exp \Big (\frac{q}{p} \int _\mathbb {T}|P_N(u+ \varphi )|^p dx 1\!\!1_{ \Vert P_N(u+ \varphi ) \Vert _{L^2}^2 \le K}\Big ) \Big ] < \infty . \end{aligned}$$

Since $\varphi \in H^1$, because of the Sobolev embedding $H^1 \subset L^p$, it is enough to show that for every β>0, $\widetilde{K} > 0$, we have that

$$\begin{aligned} \mathbb {E}_{\mu }\Big [ \exp \Big ( \beta \int _\mathbb {T}|u|^p dx 1\!\!1_{ \Vert u \Vert _{L^2}^2 \le \widetilde{K}}\Big ) \Big ] < \infty . \end{aligned}$$

This however follows from [33, Proposition 3.1]. □

### Lemma 4.3

Let 4<p<6. Let M≥1 be a dyadic number, and suppose that $\varphi \in H^1(\mathbb {T})$ satisfies

$$\begin{aligned} \Vert \varphi \Vert _{H^1} \lesssim M, \quad \Vert \varphi \Vert _{L^2}^2 \le K/2, \quad \Vert \varphi \Vert _{L^{p-2}}^{p-2} \gtrsim M^{\frac{p}{2} - 2}, \end{aligned}$$

and

4.5 $$\begin{aligned} \Vert \varphi \Vert _{L^p}^p \sim M^{\frac{p}{2} - 1}. \end{aligned}$$

Let $0 < \varepsilon _0 \ll 1$, $N \ge N_0(\varphi )$ be big enough, and let $\Theta _1$ be a 1-optimiser for the expression

4.6 $$\begin{aligned} \begin{aligned}&\log \widetilde{Z_{N,M,\varphi }} \\&= \sup _{\Theta \in \mathbb {H}_a} \mathbb {E}\bigg [ \frac{1}{p}\int _\mathbb {T}|P_N Y_1 + P_N \varphi + P_N \Theta (1)|^{p} dx 1\!\!1_{\Vert P_N Y_1 + P_N \varphi + P_N \Theta (1) \Vert _{L^2}^2 \le K}\\&\phantom {= \sup \mathbb {E}\bigg [ ]} - \varepsilon _0 M^{\frac{p}{2}-1} 1\!\!1_{\Vert P_N Y_1 + P_N \varphi + P_N \Theta (1) \Vert _{L^2}^2 > K}\\&\phantom {= \sup \mathbb {E}\bigg [ ]} - \frac{1}{2} \int _0^1 \Vert \dot{\Theta }(t) \Vert _{H^1_x}^2 dt \bigg ], \end{aligned} \end{aligned}$$

where $Y_1$ is defined as in (2.5). Then

4.7 $$\begin{aligned} \log \widetilde{Z_{N,M,\varphi }} \gtrsim M^{\frac{p}{2} -1} \end{aligned}$$

and

4.8 $$\begin{aligned} \mathbb {E}\Big [\int _\mathbb {T}|P_N \varphi + P_N \Theta _1(1)|^{p-2} dx 1\!\!1_{\Vert P_N Y_1 + P_N \varphi + P_N \Theta _1(1) \Vert _{L^2}^2 \le K}\Big ] \gtrsim M^{\frac{p}{2} - 2}. \end{aligned}$$

### Proof

First of all, by choosing $\Theta \equiv 0$, we have from (4.5) that

$$\begin{aligned} \log \widetilde{Z_{N,M,\varphi }} \gtrsim M^{\frac{p}{2} - 1}, \end{aligned}$$

which is (4.7). Now suppose by contradiction that for some constant $\delta _0 \ll 1$ small enough (to be determined later),

4.9 $$\begin{aligned} \mathbb {E}\Big [\int _\mathbb {T}|P_N \varphi + P_N \Theta _1(1)|^{p-2} dx 1\!\!1_{\Vert P_N Y_1 + P_N \varphi + P_N \Theta _1(1) \Vert _{L^2}^2 \le K}\Big ] \le \delta _0 M^{\frac{p}{2} - 2}. \end{aligned}$$

By Gagliardo-Nirenberg, we have that

$$\begin{aligned} \Vert P_N \Theta _1(1) \Vert _{L^p}^p \lesssim \Vert P_N \Theta _1(1) \Vert _{L^2}^{\frac{p}{2} +1 }\Vert P_N \Theta _1(1) \Vert _{H^1}^{\frac{p}{2} - 1}. \end{aligned}$$

Define the set

$$\begin{aligned} E_< := \{\Vert P_N Y_1 + P_N \varphi + P_N \Theta _1(1) \Vert _{L^2}^2 \le K\}, \end{aligned}$$

and let $E_>$ be its complement. On $E_<$, for some constant C=C(p,K), by Young’s inequality we have that

$$\begin{aligned} \Vert P_N \Theta _1(1) \Vert _{L^p}^p \le C (1 + \Vert P_N Y_1\Vert _{L^2})^\frac{2p+4}{6-p} + \frac{1}{8} \Vert P_N \Theta _1(1) \Vert _{H^1}^2, \end{aligned}$$

which implies that

4.10 $$\begin{aligned} \begin{aligned}&\frac{1}{p}\int _\mathbb {T}|P_N Y_1 + P_N \varphi + P_N \Theta _1(1)|^{p} dx 1\!\!1_{E_<} \\&\le C \big (1+ \Vert P_N Y_1 \Vert _{L^p}^p + \Vert \varphi \Vert _{L^p}^p + \Vert P_N Y_1\Vert _{L^2}^\frac{2p+4}{6-p}\big )1\!\!1_{E_<} \\&\qquad + \frac{1}{8} \Vert P_N \Theta _1(1) \Vert _{H^1}^2 1\!\!1_{E_<} \end{aligned} \end{aligned}$$

and proceeding similarly with p-2 instead of *p*, we have

4.11 $$\begin{aligned} \begin{aligned} \Vert P_N \Theta _1(1) \Vert _{L^{p-2}}^{p-2}&\le \varepsilon _0 M^\frac{2p-8}{8-p} (1 + \Vert P_N Y_1\Vert _{L^2})^\frac{2p}{8-p} \\&\qquad + C M^{-2} \Vert P_N \Theta _1(1) \Vert _{H^1}^2. \end{aligned} \end{aligned}$$

Since $\Vert \varphi \Vert _{L^{p-2}}^{p-2} \gtrsim M^{\frac{p}{2} - 2 }$, when $N \ge N_0 (\varphi )$ is big enough, on the set

$$ G_{<} := \{ \int _\mathbb {T}|P_N \varphi + P_N \Theta _1(1)|^{p-2} dx \le \varepsilon _0 M^{\frac{p}{2}-2} \} \cap E_{<}, $$

we must have that $\Vert P_N \Theta _1(1)\Vert _{L^{p-2}}^{p-2} \gtrsim M^{\frac{p}{2}-2}$ as well,

and so for *M* big enough, we have from (4.11) that

4.12 $$\begin{aligned} \mathbb {E}\Big [\Vert P_N \Theta _1(1) \Vert _{H^1}^2 1\!\!1_{G_<}\Big ] \ge C M^{2} \mathbb {P}(G_<) - C \varepsilon _0 M^\frac{2p-8}{8-p} \end{aligned}$$

provided that 4<p<6. Moreover, by Gagliardo-Nirenberg inequality, we have that

4.13 $$\begin{aligned} \Vert u\Vert _{L^p}^p \lesssim \Vert u \Vert _{H^1}^{\frac{4}{p} } \Vert u\Vert _{L^{p-2}}^\frac{p^2 - 4}{p} \le \frac{1}{4} \Vert u \Vert _{H^1}^2 + C \Vert u\Vert _{L^{p-2}}^{p+2}. \end{aligned}$$

In particular, by (4.13) and the definition of $G_<$, we obtain that

4.14 $$\begin{aligned} \begin{aligned} \mathbb {E}\Big [\int _\mathbb {T}|P_N \varphi + P_N \Theta _1(1)|^{p} dx 1\!\!1_{G_<}\Big ]&\le \frac{1}{4} \mathbb {E}\Big [\Vert P_N \Theta _1(1) \Vert _{H^1}^2 1\!\!1_{G_<}\Big ] \\&\quad + C M^{\frac{(p+2)(p-4)}{2(p-2)}} \mathbb {P}(G_<). \end{aligned} \end{aligned}$$

Since 2<p<6, note that

$$\begin{aligned} \frac{(p+2)(p-4)}{2(p-2)} < 2. \end{aligned}$$

Note also that by (4.9), we must have that

4.15 $$\begin{aligned} \mathbb {P}(E_<\setminus G_<) \le \delta := \min \big (\frac{\delta _0}{\varepsilon _0 \mathbb {P}(E_<)},\mathbb {P}(E_<)\big ) \le \sqrt{\frac{\delta _0}{\varepsilon _0}}, \end{aligned}$$

and so

$$\begin{aligned} \mathbb {P}(G_<) + \mathbb {P}(E_>) \ge 1 - \delta . \end{aligned}$$

Therefore, by (4.6), (4.7), (4.9), (4.10), (4.14), we deduce that

$$\begin{aligned}&\log \widetilde{Z_{N,M,\varphi }} \\&\begin{aligned} \le 1 + \mathbb {E}\bigg [ \frac{1}{p}\int _\mathbb {T}|P_N Y_1 + P_N \varphi + P_N \Theta _1(1)|^{p} dx 1\!\!1_{\Vert P_N Y_1 + P_N \varphi + P_N \Theta _1(1) \Vert _{L^2}^2 \le K}\\ \hspace{40pt}- \varepsilon _0 M^{\frac{p}{2}-1} 1\!\!1_{\Vert P_N Y_1 + P_N \varphi + P_N \Theta _1(1) \Vert _{L^2}^2> K} - \frac{1}{2} \int _0^1 \Vert \dot{\Theta }_1(t) \Vert _{H^1_x}^2 dt \bigg ]. \end{aligned} \\&\begin{aligned} \le C(1 + M^{\frac{p}{2} - 1} \mathbb {P}(E_< \setminus G_<) + \varepsilon _0 M^\frac{2p-8}{8-p}) \\ \hspace{80pt}+ \mathbb {E}\Big [- \varepsilon _0 M^{\frac{p}{2} - 1} 1\!\!1_{E_>} - \frac{1}{8} \Vert P_N \Theta _1(1) \Vert _{H^1}^2 \Big ] \end{aligned} \\&\le 2C \delta M^{\frac{p}{2} -1} - \varepsilon _0 (\mathbb {P}(G_<) + \mathbb {P}(E_>))M^{\frac{p}{2} -1}, \end{aligned}$$

which is a contradiction with (4.7) when $\delta \ll 1$ is small enough, which in view of (4.15), is the case as soon as $\delta _0$ in (4.9) is small enough.

□

### Proof of Theorem 1.4

Suppose that $\varphi _M$ satisfies the hypotheses of Lemma 4.3. Such a family of $\varphi _M$’s can be built (for instance) by choosing $\varphi _1$ to be a nonnegative smooth function with compact support with $\Vert \varphi _1 \Vert _{L^{2}}^2 \le K/2$, and

$$\begin{aligned} \varphi _M(x) = M^{\frac{1}{2}} \varphi _1(Mx). \end{aligned}$$

Recall the probability measures $\rho _{N,L,\varphi }$ introduced in (4.2). With this notation, we have that

4.16 $$\begin{aligned} \begin{aligned}&d\rho _{N,\varepsilon _0M^{\frac{p}{2}-1},\varphi } (u)\\&\quad \quad := \frac{1}{\widetilde{Z_{N,M,\varphi }}}\exp \Big (\frac{1}{p} \int _\mathbb {T}|P_N(u+\varphi )|^p dx 1\!\!1_{\Vert P_N(u+\varphi ) \Vert _{L^2}^2 \le K} \\&\quad \quad \quad \quad \quad \quad \quad \quad \quad \quad - \varepsilon _0 M^{\frac{p}{2} -1}1\!\!1_{\Vert P_N(u+\varphi ) \Vert _{L^2}^2 > K} \Big ) d \mu (P_Nu), \end{aligned} \end{aligned}$$

and analogously

4.17 $$\begin{aligned} \begin{aligned}&d\rho _{\infty ,\varepsilon _0M^{\frac{p}{2}-1},\varphi } (u)\\&\quad = \frac{1}{\widetilde{Z_{M,\varphi }}}\exp \Big (\frac{1}{p} \int _\mathbb {T}|u+\varphi |^p dx 1\!\!1_{\Vert u+\varphi \Vert _{L^2}^2 \le K} \\&\qquad \phantom {XXXXXXXXX}- \varepsilon _0 M^{\frac{p}{2} -1}1\!\!1_{\Vert u+\varphi \Vert _{L^2}^2 > K} \Big ) d \mu (u). \end{aligned} \end{aligned}$$

Note that the definition of $\widetilde{Z_{N,M,\varphi }}$ in (4.16) coincides with the expression (4.6) in view of Lemma 2.4.

Then, by Lemma 2.5, (4.8), and monotone convergence theorem, we have that

$$\begin{aligned} \mathbb {E}_{\rho _{N,\varepsilon _0M^{\frac{p}{2}-1},\varphi }}\Big [\int _\mathbb {T}|u|^{p-2} dx 1\!\!1_{\Vert u\Vert _{L^2}^2 \le K} \Big ] \gtrsim M^{\frac{p}{2} -2} - C \mathbb {E}_{\mu }\big [\Vert u\Vert _{L^{p-2}}^{p-2}\big ] \gtrsim M^{\frac{p}{2} -2}. \end{aligned}$$

In view of (4.3), we can take limits as $N \rightarrow \infty ,$ and obtain that

4.18 $$\begin{aligned} \mathbb {E}_{\rho _{\infty ,\varepsilon _0M^{\frac{p}{2}-1},\varphi }}\Big [\int _\mathbb {T}|u|^{p-2} dx 1\!\!1_{\Vert u\Vert _{L^2}^2 \le K} \Big ] \gtrsim M^{\frac{p}{2} -2} \end{aligned}$$

as well. Therefore, we have that

$$\begin{aligned} \mathbb {E}_{{\rho _\varphi }}\Big [\int _\mathbb {T}|u|^{p-2}dx \Big ]&= \mathbb {E}_{\rho _{\infty ,\varepsilon _0M^{\frac{p}{2}-1},\varphi }}\Big [\int _\mathbb {T}|u|^{p-2}dx 1\!\!1_{\Vert u\Vert _{L^2}^2 \le K} \Big ]\frac{\widetilde{Z_{M,\varphi }}}{Z_\varphi }\\&\ge \mathbb {E}_{\rho _{\infty ,\varepsilon _0M^{\frac{p}{2}-1},\varphi }}\Big [\int _\mathbb {T}|u|^{p-2} dx 1\!\!1_{\Vert u\Vert _{L^2}^2 \le K} \Big ]\\&\gtrsim M^{\frac{p}{2} -2}, \end{aligned}$$

where we used (4.18) in the last step. This shows that

$$\begin{aligned} \sup _{\varphi \in H^1} \mathbb {E}_{\rho _\varphi }\Big [\int _\mathbb {T}|u|^{p-2} dx \Big ] \gtrsim M^{\frac{p}{2} -2}. \end{aligned}$$

Since *M* is arbitrary, we obtain (1.16). Together with (1.15), we obtain (1.17). □

## Footnotes

1 By convention, we normalize $B_n$ so that $\text {Var}(B_n(t)) = t$.
